# 

**Published:** 1891-03

**Authors:** 


### CONTENTS:

Article I.—Pyorrhea Alveolaris. Ay B. F. Arrington, M.D., D D.S. - - - 481

" II.—The Application af Massage in the Treatment of Dental Pathological Conditions. By W. F. Rehfuss, D.D.S. - - - - 492

" III.—A Review of Copper Amalgam. By Julien W. Russell, M.D.S. - - - - 498

" IV.—Broken Instruments in Root Canals. By Wm. Conrad, D.D.S. - - - 503

" V.—Adhesion of Soft Palate to Pharynx , Operation ; Cure. By Hal Foster, M. D. - - - 507

" VI.—Some Practical Suggestions about Plaster of Paris. By Rodrigues Ottolengui. - - - 510

" VII.—The Dental Pulp. By W. Xavier Sudduth, A.M., M.D., D.D.S. - - - - 515

### COMMUNICATIONS.

Missouri Dental College. - - - - - 516

Missouri Dental College Alumni. - - - - 519

Illinois State Dental Society. - - - - - 520

### BIBLIOGRAPHICAL.

Harris' Dictionary. - - - - - - 522

### MONTHLY SUMMARY.

Hæmorrhage after Tooth Extraction and the Immediate Treatment 525

Dental Prosthesis. - - - - - . 527

Teeeth in Diabetes. - - - - - - 528

